# Study on the Mechanical Properties of 3D-Printed Sand Mold Specimens with Complex Hollow Structures

**DOI:** 10.3390/ma17050996

**Published:** 2024-02-21

**Authors:** Jingying Xu, Jinwu Kang, Yongkang Hu, Houfa Shen, Weimin Mao

**Affiliations:** 1 School of Materials Science and Engineering, University of Science and Technology Beijing, Beijing 100083, China; 18689840791@163.com (J.X.); mao_wm@ustb.edu.cn (W.M.); 2 Key Laboratory for Advanced Materials Processing Technology, School of Materials Science and Engineering, Tsinghua University, Beijing 100084, China; huykchn@gmail.com (Y.H.); shen@tsinghua.edu.cn (H.S.)

**Keywords:** 3D printing, hollow sand mold, casting, mechanical property, multi-layer shell

## Abstract

Casting, as a fundamental process in metal forming, finds widespread applications in the manufacturing industry. The advent of 3D printing hollow sand mold technology presents a novel method for casting technology to revolutionize traditional dense sand molds, offering increased flexibility in achieving quality control and improvement in casting processes. Consequently, this study delves into an examination of the mechanical strengths of 3D-printed sand molds with complex hollow structures and further investigates the influence of hollow sand mold concession on castings. The results indicate that compressive and high-temperature residual tensile and bending strengths vary in hollow structures. Multi-layer shells have greater high-temperature residual tensile, compressive, and bending strengths than truss hollow sand molds with roughly the same hollow volume fraction. Compared to dense sand molds, hollow sand molds, which have a lower mechanical strength, have better retractability, which helps reduce the residual stress and crack tendency of castings. The breaking of hollow structures is limited to local areas, unlike the penetrative cracking of dense sand molds. The I-beam-shaped casting test results indicate that a hollow structure is beneficial for the preservation of the integrity of a sand mold during the casting process. Compared to dense and truss hollow molds, a multi-layer shell hollow sand structure has the comprehensive advantages that it improves retractability while maintaining strength relatively well, reduces the residual stress, and avoids cracks in castings and itself.

## 1. Introduction

Sand casting is one of the basic metal processing methods, extensively employed in sectors such as aerospace, aeronautics, power equipment, automobiles, mechanical manufacturing, etc. [1,2,3,4]. Currently, there is a growing demand for high-end castings such as single-crystal blades, rocket barrel bodies, missile hulls, etc. [5,6]. However, traditional sand molds typically have a dense structure that is thick and bulky, thereby impacting the casting process and fine cooling control. Three-dimenionalprinting sand mold technology presents a superior process option for producing intricate, high-quality castings. A hollow sand structure outperforms conventional dense sand structures with its flexible design capability, enabling closed-loop cooling control during the casting process, which ultimately enhances the performance of the castings. 

The properties of sand molds and cores play a crucial role in influencing both the casting process and quality. Previous studies have predominantly focused on the impact of material and printing processes on the mechanical properties of 3D-printed sand molds, and the structure of these sand molds is typically designed to be dense. Gao et al. [7] investigated the effects of X-axis resolution, layer thickness, and re-coater speed on the strength of 3D-printed sand molds. The findings revealed that as the layer thickness decreases, the porosity of the sand mold decreases, leading to an increase in the number of bonding bridges between sand particles and consequently enhancing the strength of the sand mold. Coniglio et al. [8] found that the re-coater speed is crucial for connecting all sand grains and void areas, thereby increasing the overall strength of the sand mold. Dana et al. [9] observed cross-linked resin bridges between sand particles by SEM analysis, establishing a relationship between the microstructure of the samples and the process parameters. Bobrowski et al. [10] found that the binder content had the most significant effect on the thermal deformation of the sand mold, and the deformation of the sample could be reduced by a factor of two by reducing the usage of the binder to half. Son et al. [11] investigated the impact of a curing agent and binder on the strength of 3D-printed sand molds, identifying the rate of curing reaction and the number of bonding bridges as key mechanisms. Increasing the amount of curing agent from 0.15 to 0.25 wt% resulted in an increase in the bending strength of the sand mold. However, a further increase has no effect. Primkulov et al. found that the compressive strength and stiffness increased over a short period of time after curing [12]. Additionally, a curing temperature higher than 180 °C resulted in the degradation of furan resin and a reduction in the strength of the 3D-printed sand mold [13]. The bending strength of the sand mold demonstrated an increase with higher levels of binder addition [14]. Nevertheless, excessive amounts of curing agent and binder can lead to severe degradation and erosion in 3D-printed sand molds [11]. This is attributed to the rapid curing rate and uneven polymerization caused by aggregation. Strength and gas permeability can be competing factors. Excessive amounts of binder produce too much gas during the casting process, resulting in increased casting porosity. Sivarupan et al. [15,16] used laminated volumetric images to define a 3D mesh for permeability calculations. Due to the low X- and Y-resolution of the furan droplets, the Z-direction permeability was consistently lower than the X-/Y-direction permeability. Guo et al. [17] used standard test methods to investigate the gas permeability of 3D-printed sand molds. With an increase in the curing agent content, the gas permeability of the sand molds increased. A good gas permeability reduces the metal filling resistance and can reduce the porosity defects on the surface, thus improving the quality of the castings. Wang et al. [18] compared the exhaust speed of blind and hollow sand cores and found that the peak speed of the former was 1.10 m/s, which is lower than that of the latter, 1.89 m/s.

Moreover, scholars have recently initiated studies on hollow sand molds, investigating the correlation between hollow structures and the performance of 3D-printed sand molds. Kang [19] introduced the concept of a hollow sand mold design based on 3D printing, and truss, reinforcement, and multi-layer shell hollow sand molds were designed. In the casting process, areas prone to stress concentration, such as sharp corners in truss hollow sand molds, were found to be susceptible to cracks. Therefore, adopting rounded excesses in the design of hollow sand molds proved effective in reducing the occurrence of cracks. Furthermore, a cavity in multi-layer shell hollow sand molds prevents the transfer of heat and stress to the outer shells. The lower temperature of the outer shells helps to maintain higher strengths, consistently restraining the inner shells and diminishing the tendency of cracking in the sand molds themselves [20].

However, there are currently significant gaps in understanding the mechanical properties of hollow sand molds, as well as the alterations in the properties of the sand molds themselves during the casting process. These gaps necessitate resolution through a series of casting processes and intrinsic scientific problems. Therefore, in this paper, utilizing the self-developed FT-Hollow v3.2 software, we designed hollow sand molds with different structures, including multi-layer shell and truss structures. We investigated their mechanical strengths, such as compressive strength, bending strength, tensile strength, etc. In addition, the influence of the concessional property of hollow sand molds on the deformation and residual stress of castings was studied using a pouring experiment.

## 2. Materials and Methods

### 2.1. Test Method for Mechanical Properties of Sand Mold

**Figure 1:**
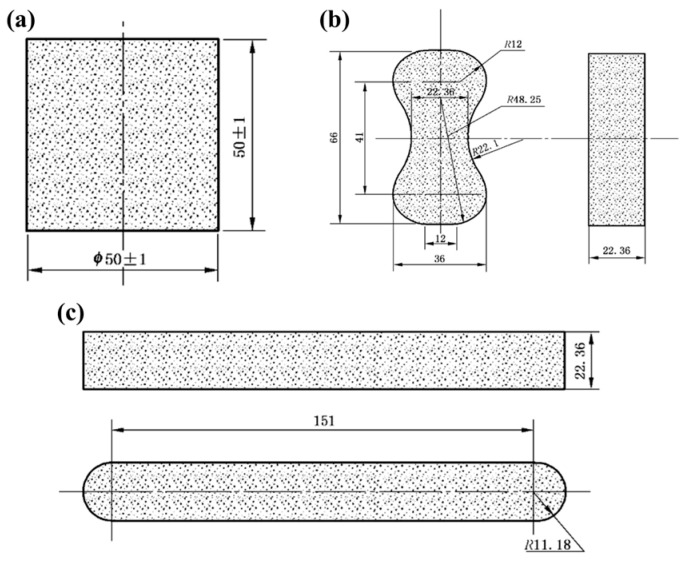
Shape and size of experimental specimens in (**a**) compressive strength test, (**b**) tensile strength test, and (**c**) bending strength test (unit: mm).

In this study, the strength of sand molds was evaluated through compressive, bending, and high-temperature residual tensile strength tests, utilizing three specimens, as illustrated in Figure 1. The compressive strength test employed standard cylindrical specimens, the bending strength test utilized elongated strip specimens, and the tensile strength test involved dog-bone-shaped specimens. The testing apparatus employed was the E45.105 Microcomputer-Controlled Electronic Universal Testing Machine, manufactured by MTS (Eden Prairie, MN, USA), a U.S. company.

**Figure 2:**
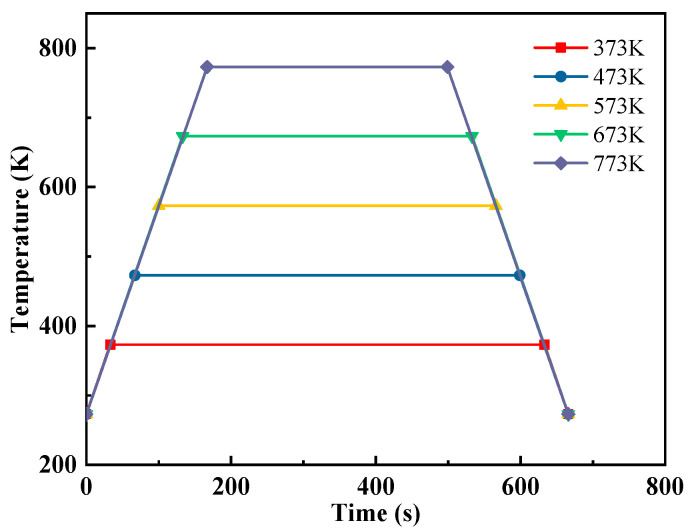
Heating temperature curve of the sand specimen.

Over the course of the high-temperature residual tensile strength test, the 3D-printed dog bone sand specimen undergoes initial heating treatment within a resistance furnace. The heating temperatures are precisely controlled at 373 K, 473 K, 573 K, 673 K, and 773 K, with a set heating rate of 3 K/s. Once the furnace temperature attains the specified level, it is maintained for 600 s to ensure uniform heating throughout the sand specimen, as illustrated in Figure 2. Subsequent to insulation, the sand specimen is removed from the resistance furnace and allowed to undergo natural cooling at room temperature. Upon cooling to room temperature, the tensile strength test is conducted to characterize the high-temperature residual tensile strength of the sand specimen.

### 2.2. Design of Hollow Sand Mold Test Specimens

**Figure 3:**
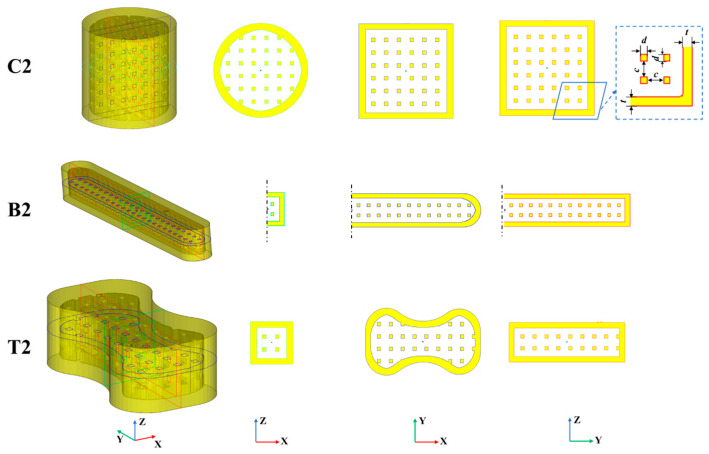
A diagram of specimen No. 2 and its center section.

**Figure 4:**
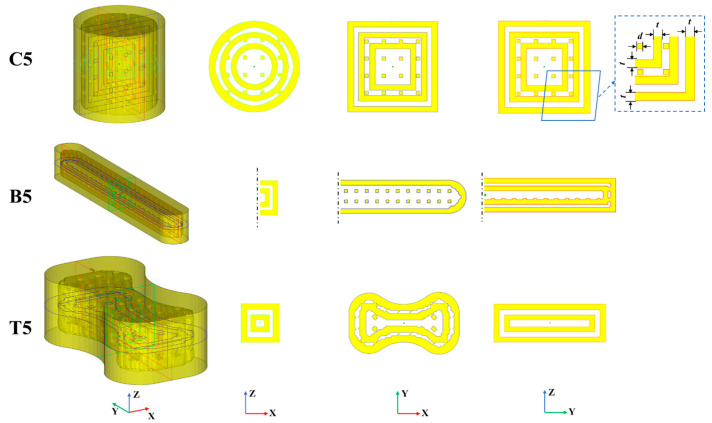
A diagram of specimen No. 2 and its center section.

In this study, FT-Hollow (a software developed by us) [19] was used to design the strength-testing sand mold specimens, consisting of an outer shell and an inner support structure. Five sets of specimens were designed for each of the three tests: compressive, bending and, tensile strength. Table 1 lists the hollow types and design parameters of these sand mold specimens. As an example, Figure 3 and Figure 4 show the internal structure of the specimen for the Truss No. 2 and Multi-layer Shell No. 5 specimens. Specimen No. 1 is dense for the control experiments. Specimen Nos. 2, 3, and 4 are truss hollow structures; the spacing between trusses (*c*) is 5 mm, but the thickness of the outer shell (*t*) of the specimens and the cross-section size of the trusses (*d*) are different. Specimen No. 5 has a multi-layer shell structure with a thickness of 3 mm for each layer, and the shells are connected to one another by a truss support structure. It is worth noting that due to the limited height of the bending and tensile specimens, the number of shells in the vertical direction of specimen Nos. B5 and T5 is set to 2, and the number of shells in the vertical direction of the compressive specimen No. C5 is set to 3.

Note that for the compressive and bending specimens, the hollow volume fraction is the volume rate, while for the tensile specimens, the hollow volume fraction is the hollow volume fraction of the middle section perpendicular to the tensile direction.

### 2.3. Three-Dimensional Printing of Hollow Sand Specimens

**Figure 5:**
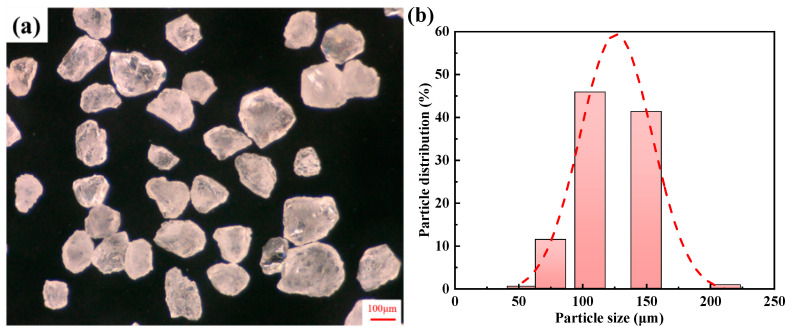
The particle morphology and particle size range of silica sand grains. (**a**) Particle morphology; (**b**) particle size range.

These sand molds were 3D printed with an ExOne Smax 3D Printer, which is produced by the German company ExOne (Gersthofen, Germany). Firstly, the sand grains were mixed with a curing agent, then the curing agent-coated sand grains were sequentially spread over the worktable, and afterward, the binder was injected through a printing head that consists of hundreds of tiny nozzles, following the profile of each section of the mold. This printing process occurred layer by layer until the mold was complete, with a printed layer thickness of 0.2 mm. The original sand utilized in this experiment is silica sand, with a purity of approximately 99.4% SiO_2_ and low humidity. The particle morphology and particle size range of the silica sand grains used for printing are illustrated in Figure 5. The sand grain size is mainly distributed in a range of 100–150 μm, and the shape is irregular. The binder employed in this experiment was a furan resin binder, with a spraying amount equivalent to about 1.6~1.8% of the weight of the silica sand. The curing agent was a sulfonic acid curing agent, comprising approximately 0.2% of the weight of the silica sand. It should be noted that the loose sand within the hollow part of the hollow sand mold was not cleaned during the strength test.

## 3. Results and Discussion

### 3.1. Compressive Strength of Hollow Sand Mold

**Figure 6:**
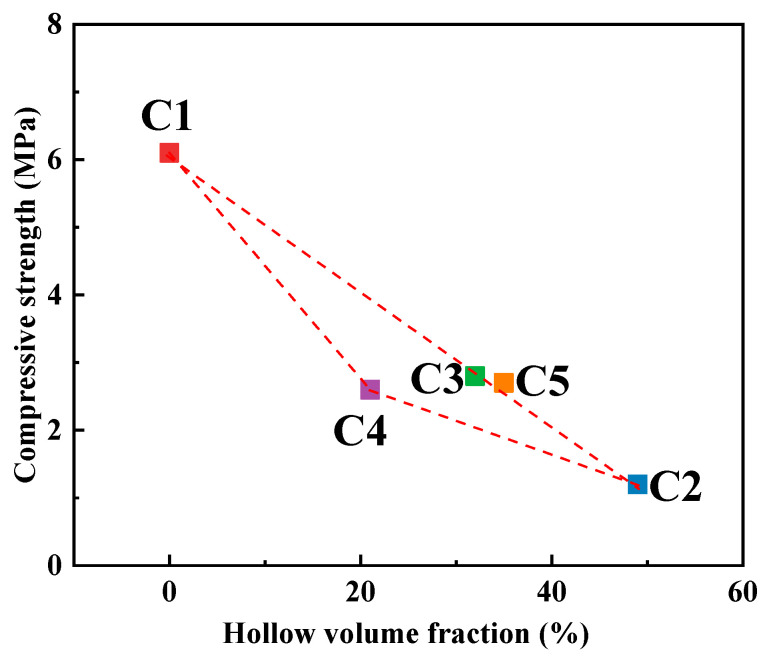
Relation curve between hollow volume fraction and compressive strength of sand mold specimens.

The compressive strength results for the five sand molds are presented in Figure 6. From the figure, it is evident that the compressive strength of the traditional dense sand specimen (No. C1) is the highest, registering at 6.1 MPa. Conversely, the compressive strength of the hollow sand specimens experiences a significant reduction, ranging from 54% to 80%. However, the compressive strength and the hollowing rate of the hollow sand molds are not linear. Specimen No. C4 has the lowest hollow volume fraction among the hollow sand molds, and its compressive strength, at 2.6 MPa, is not the highest. The hollow volume fraction of the multi-layer shell hollow sand specimen, No. C5, is higher than that of specimen No. 3, but its compressive strength is also higher than that of truss specimen No. 3. 

In comparing the truss hollow sand specimens (Nos. C2–C4), it is observed that increasing the thickness of the outer shell significantly enhances the compressive strength when the cross-sectional size of the internal trusses remains constant. However, augmenting the cross-sectional size of the trusses has a limited effect on the compressive strength when the outer shell thickness remains constant. Additionally, a comparison between the compressive strength of the truss hollow sand specimens (Nos. C2–C4) and the multi-layer shell hollow sand specimen (No. C5) reveals that the multi-layer shell structure can further enhance the compressive strength under the condition of identical outer shell thickness and truss cross-sectional size.

**Figure 7:**
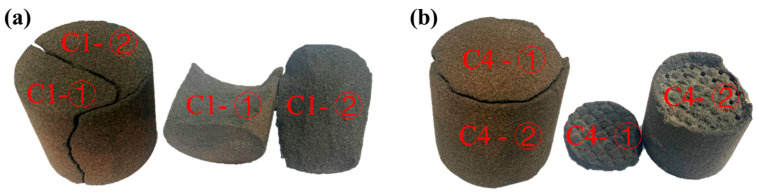
Fracture state of sand specimens in compressive test: (**a**) No. C1 and (**b**) No. C4.

Figure 7 illustrates the fracture conditions of sand specimens No. C1 and No. C4 after the compressive test. The dense sand specimen No. C1 exhibits cracking along the middle and sides of the upper surface, resulting in the specimen being bifurcated—representing a comprehensive fracture. On the other hand, the truss hollow sand specimen No. C4 experiences a split solely on the entire upper surface, with the main body of the sand sample remaining uncracked, presenting a relatively complete structure. The damage from the applied test load on the hollow sand mold is partial. This observation suggests that the compressive load does not propagate into the interior of the hollow sand specimen along the outer surface. The hollow structure effectively hinders the transfer of force from the outside to the interior of the hollow sand specimen, thereby contributing to the preservation of the hollow sand mold’s integrity during the casting process. This structural integrity makes it less susceptible to the development of penetrating cracks. Furthermore, during the test process, due to the loose sand within the hollow part not being cleaned, this loose sand demonstrated some compressive resistance.

### 3.2. Bending Strength of Hollow Sand Mold

**Figure 8:**
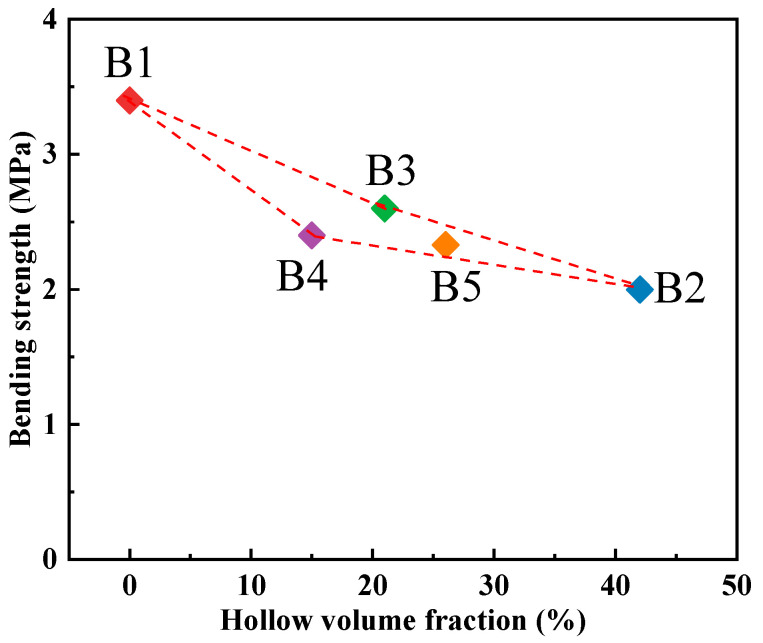
Relation curve between hollow volume fraction and bending strength of sand specimen.

The results of the bending strength assessments for the five sand specimens are presented in Figure 8. It is evident that the bending strength of the traditional dense sand specimen (No. B1) remains the highest, reaching 3.4 MPa, and the bending strength of the hollow sand specimens was reduced. However, the decrease in the bending strength of the hollow structure specimens is less significant compared to the compressive strength. The truss hollow sand specimen No. B2, featuring the highest degree of hollow volume fraction, exhibits the lowest bending strength at only 2.0 MPa. On the other hand, the bending strength of the truss hollow sand specimen No. B3 is the highest, reaching up to 2.6 MPa. Comparing the bending strength of the three groups of trussed hollow sand specimens (Nos. B2–B4), it is evident that the supporting effect of the internal truss structure on the bending strength is not as pronounced as that of the outer shell thickness.

Furthermore, comparing the bending strength of the hollow sand specimens No. B2 and No. B5 reveals that a multi-layer shell structure can enhance the bending strength of sand specimens under identical outer shell thickness and truss cross-section size conditions. Therefore, with a focus on achieving a higher hollow volume fraction, the bending strength of hollow sand can be enhanced by increasing the outer shell thickness and incorporating a multi-layer shell structure into the hollow sand specimen, as demonstrated in sand specimen No. B5.

### 3.3. High-Temperature Residual Tensile Strength Analysis of 3D-Printed Hollow Sand

The previous study found that under high-temperature conditions, the resin binder in a 3D-printed sand mold undergoes decomposition and volatilization due to heat. Consequently, the adhesion of the resin binder weakens, leading to a reduction in the strength of the sand. Therefore, further investigation into the strength and stability of 3D-printed hollow sand molds at elevated temperatures is necessary. We conducted a study on the high-temperature residual tensile strength of 3D-printed hollow sand molds.

**Figure 9:**
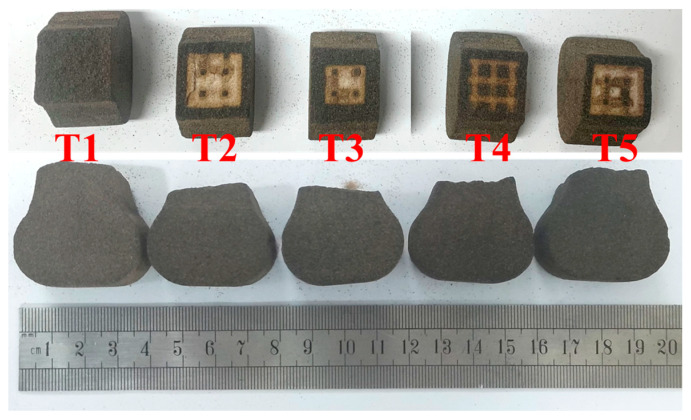
The broken sand specimens after high-temperature residual tensile test.

Figure 9 illustrates the fracture section of the sand specimens post-high-temperature residual tensile tests. It is evident that the fracture in all the sand specimens occurs in the thinnest middle section of the specimen. Therefore, the hollow volume fraction at the center section of the sand specimen is used to calculate the hollow volume fraction of the sand specimen with tensile strength.

**Figure 10:**
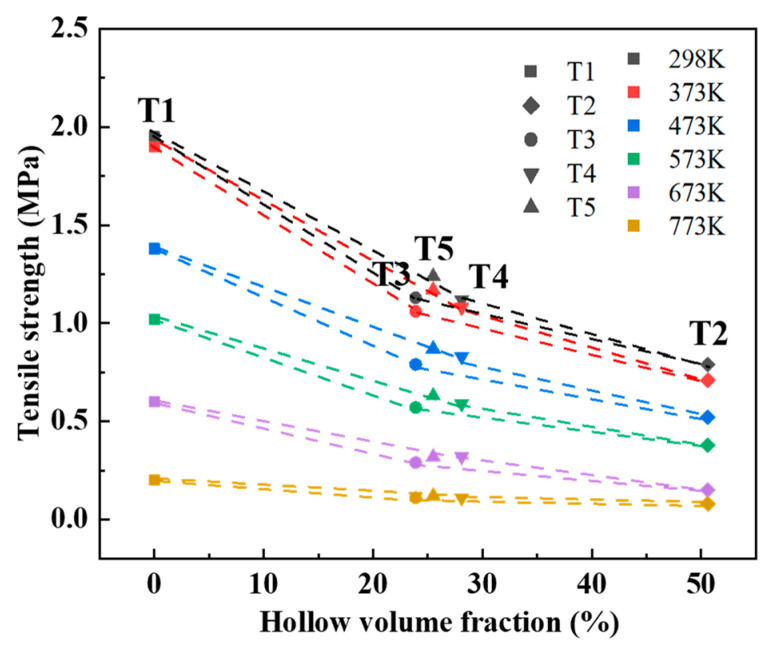
Relation curve between hollow volume fraction and high-temperature residual tensile strength of sand specimens.

Figure 10 depicts the high-temperature residual tensile strength of five sand specimens with different hollow volume fractions under varying heating temperatures. As observed in Figure 10, the high-temperature residual tensile strength decreases as the heating temperature increases. Moreover, at equivalent heating temperatures, the high-temperature residual tensile strength of dense sand specimens surpasses that of hollow sand specimens. At room temperature, the tensile strength of the dense sand specimen No. 1 is the highest at 1.95 MPa. In contrast, the room-temperature residual tensile strength of the hollow sand specimens (No. T2, No. T3, No. T4, and No. T5) is 0.79, 1.13, 1.12, and 1.24 MPa, respectively. This signifies a reduction in the residual tensile strength by 36% to 59% compared to dense sand specimens. This disparity primarily stems from the absence of a binder within the hollow part of the hollow sand molds.

Following heating at 373 K for 600 s, a slight decrease in the residual tensile strength at high temperatures is observed compared to room temperature, albeit marginal. However, as the heating temperature rises to 473 K or above, the high-temperature residual tensile strength experiences a decrement with increasing temperature. This phenomenon primarily arises due to the thermal degradation of the weak chemical bonds within the binder of the 3D-printed sand mold. The molecular chain break decomposition reaction of the furan binder occurs at approximately 453 K, and the extent of binder decomposition in the specimen amplifies with higher heating temperatures. Consequently, a decline in the residual tensile strength of the specimen at elevated temperatures is observed.

In addition, owing to the impact of bonding bridges on the tensile strength, it can be found that within the three truss hollow sand molds (Nos. T2–T4), the higher the hollow volume fraction, the lower the tensile strength. However, the multi-layer shell hollow sand mold (No. T5) does not follow this law. The hollow volume fraction of the multi-layer shell hollow sand specimen No. T5 surpasses that of the trussed hollow sand specimen No. T3. Despite this, both the room-temperature and high-temperature residual tensile strengths of sand specimen No. T5 exceed those of sand specimen No. T3. Thus, it is evident that the multi-layer shell hollow sand specimen, with an equivalent hollow volume fraction, can substantially enhance both room-temperature and high-temperature residual tensile strengths.

**Figure 11:**
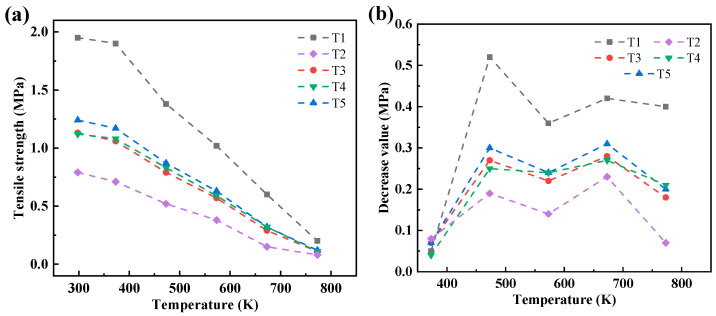
(**a**) Variation in high-temperature residual tensile strength of sand molds with temperature; (**b**) the decrease in high-temperature residual tensile strength with increasing temperature.

Figure 11 shows the decrease in the high-temperature residual tensile strength compared to the room-temperature tensile strength of the five sand specimens. The dense sand specimen exhibits the most substantial reduction in the high-temperature residual tensile strength with temperature, namely, a decrease of 0.5 MPa after heating to 473 K, while the hollow sand specimen only experiences a decrease of 0.25 MPa. As the heating temperature further increases, the disparity between the high-temperature residual tensile strength of the dense and hollow sand specimens diminishes. At 773 K, the high-temperature residual tensile strength of all five sand specimens falls below 0.2 MPa. In comparison to the dense sand molds, the high-temperature residual tensile strength of the hollow sand mold decreases less with temperature, suggesting that the hollow structure contributes to slowing down heat transfer. Leveraging this characteristic of the hollow sand mold, during the casting process, the inner shell of the sand mold, which comes into direct contact with the casting, undergoes combustion at high temperatures. Simultaneously, the outer shell of the sand mold maintains its strength, ensuring the overall integrity of the sand mold without structural failure.

Table 2 shows the ratio (R_S_) of the strength decreases of the hollow sand specimens in comparison to the dense sand specimen over the hollow volume fraction. The ratio is calculated with the formula given below. The strength value for No. 2 is considerably low, which cannot meet the strength requirements of sand mold design in the casting process. The strength of hollow sand specimens Nos. 3–5 meets the requirements. Therefore, in this comparison, we selected the truss hollow sand specimens, Nos. 3–4, and multi-layer shell sand sample No. 5 with similar hollow volume fractions. As shown in the table, the ratio for multi-layer shell sand sample No. 5 is smaller than that of the other two truss sand samples. Under the same hollow volume fraction, the multi-layer shell hollow sand specimen demonstrates a significant improvement in both strengths for the sand samples.

$$R_{S}=\frac{S_{d}-S_{h}}{S_{d}\;*\;h}$$

where $S_{d}$ is the strength of a dense sand specimen, $S_{h}$ is the strength of a hollow sand specimen, and *h* is the hollow volume fraction.

### 3.4. Influence of Sand Mold Concession on Castings

During the solidification process of metal castings, the sand mold can retard the shrinkage of the casting, and its expansion when heated will further enhance the resistance. This resistance induces a stress concentration within or on the surface of the casting, potentially resulting in deformation or cracking. In the industry, the retractability of sand molds is usually described by their bending and compressive strengths. Higher values of bending strength and compressive strength in the sand mold indicate poorer retractability. Consequently, in comparison to dense sand molds, although the bending strength and compressive strength of hollow sand molds are diminished, their retractability is enhanced. This improvement in concession leads to a reduction in residual stress within the casting, contributing to a more favorable environment for preventing deformation or cracking of the casting.

**Figure 12:**
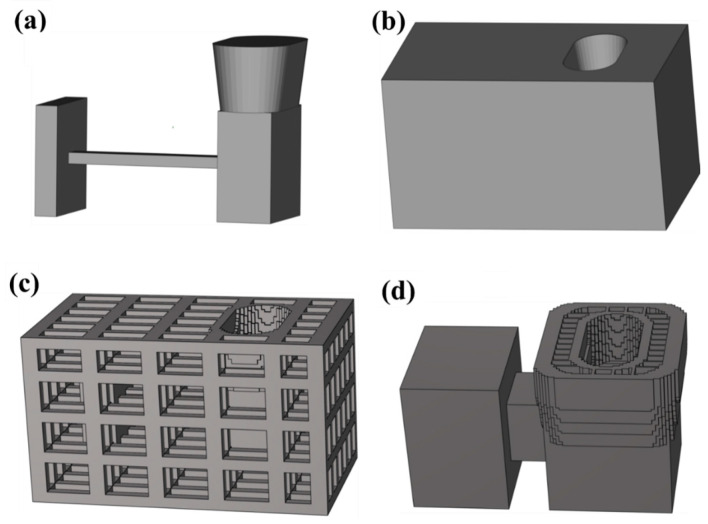
The design of casting and sand molds: (**a**) casting, (**b**) P1DN, (**c**) P2TN, and (**d**) P3SN [20].

**Figure 13:**
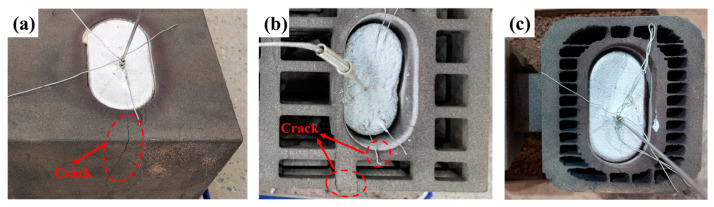
The cracks in the sand molds: (**a**) P1DN, (**b**) P2TN, and (**c**) P3SN [20].

In our preceding research, “I-beam” castings were designed, and three sand molds—namely, a traditional dense sand mold (P1DN), a truss hollow sand mold (P2TN), and a multi-layer hollow sand mold (P3SN)—were fabricated using 3D printing technology, as depicted in Figure 12 [20]. It was observed that, in the absence of sand box constraints, during the casting process, the dense sand mold exhibited more significant cracks, and the truss hollow sand mold also had cracks, whereas the multi-layer shell hollow sand mold displayed no evident cracks, as shown in Figure 13. A multi-layer shell structure can prevent heat transfer from the inner shell to the outer shell, maintain high strength, and consistently impose constraints on the inner shell, thereby reducing the tendency of the sand mold to crack. The multi-layer shell hollow sand mold demonstrated superior retractability properties.

**Figure 14:**
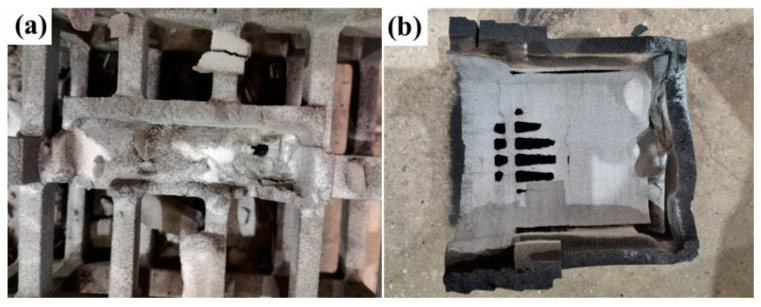
Burnout of sand molds: (**a**) P2TN and (**b**) P3SN.

Following the shakeout treatment of the castings, we obtained the sand molds post-shakeout, as illustrated in Figure 14. Owing to the high temperature of the casting, the binder in the sand mold part near the casting was entirely burned off. Consequently, this part of the sand automatically collapsed, resulting in a lighter sand color. However, the outermost part of the sand mold remained essentially unchanged, showing no signs of burning.

To further substantiate the improved retractability of the hollow sand molds, the linear contraction and residual stress of the thin rod in the middle of the castings obtained from three kinds of sand molds were tested. I residual stresses of the specimens were measured with a Spider X-EDGE portable theta diffractometer (GNR, Torino, Italy) using the X-ray diffraction method. Table 3 presents data on the linear contraction and residual stress of the central thin rod in A357 aluminum alloy castings. As observed in Table 3, the central thin rod in all three casting groups experiences contraction, with the castings of dense sand mold (P1DN) exhibiting the least linear contraction. This phenomenon is attributed to the poor retractability of dense sand molds, resulting in the most significant hindrance to casting shrinkage, thereby minimizing shrinkage deformation and causing the highest residual stress within the castings—measuring at 43.65 MPa. Consequently, the likelihood of internal casting cracks is increased. In contrast, the hollow sand mold exhibits improved retractability, leading to reduced hindrance to casting shrinkage. Although the linear contraction of the castings with the hollow sand mold is greater than that of the dense sand mold, the residual stress within the castings is diminished. In particular, the residual stress in the casting of the multi-layer shell hollow sand mold (P3SN) is the lowest at 9.29 MPa, representing a reduction of approximately 79%. This substantial reduction in residual stress significantly decreases the probability of cracks occurring in the castings.

## 4. Conclusions

In this study, hollow sand molds with various structures were designed by the self-developed professional software FT-Hollow, including multi-layer shell and truss structures. Mechanical strengths, including compressive, bending, and high-temperature residual tensile, were investigated. Additionally, the impact of the hollow structure on the sand mold’s concessional properties and its own crack tendency was further explored through experiments. The main conclusions are as follows:

1. The multi-layer shell hollow sand structure has higher compressive, high-temperature residual tensile, and bending strengths compared to the truss structure with similar hollow volume fractions. It has the lowest ratio strength decrease over hollow volume fraction. Compared to dense structures, the low strengths provide the hollow structure with improved retractability, which is beneficial as it helps reduce residual stress and avoid cracks in castings.
2. The compression failure of the hollow structure results in surface fractures, while the dense sand mold undergoes penetrating fractures. Consequently, the hollow structure is less prone to penetration cracks. The I-beam-shaped casting test results indicate a significant crack in the dense sand mold and a local crack in the truss hollow sand mold, whereas there was no crack in the multi-layer shell hollow sand mold.
3. The I-beam-shaped casting experiments confirmed the decrease in residual stress in the castings with a hollow sand mold.
4. The high-temperature residual tensile strength of a dense sand specimen decreases significantly with temperature compared to that of hollow sand specimens. The insulation effects of hollow structures can retard heat transfer in hollow sand molds, which helps in maintaining temperature at a low level and preserving the initial strength of the outer shell.
5. Compared to dense and truss hollow molds, the multi-layer shell hollow sand structure has comprehensive advantages in that it improves retractability while maintaining strength relatively well, reduces the residual stress, and helps avoid cracks in the castings and itself. It is suggested for future applications in casting production.

## Figures and Tables

**Table 1:** The dimensions of strength test specimens.

	No.	Hollow Type	Outer Shell Thickness (*t*) (mm)	Truss Cross-Section Size (*d*) (mm)	Hollow Volume Fraction (%)
Compressive strength	C1	Dense	-	-	0
	C2	Truss	3	2 × 2	49
	C3	Truss	5	2 × 2	33
	C4	Truss	3	4 × 4	21
	C5	Multi-layer	3	2 × 2	32
Bending strength	B1	Dense	-	-	0
	B2	Truss	3	2 × 2	42
	B3	Truss	5	2 × 2	21
	B4	Truss	3	4 × 4	15
	B5	Multi-layer	3	2 × 2	26
High-temperature residual tensile strength	T1	Dense	-	-	0
	T2	Truss	3	2 × 2	51
	T3	Truss	5	2 × 2	24
	T4	Truss	3	4 × 4	28
	T5	Multi-layer	3	2 × 2	26

**Table 2:** The ratio of the strength decreases of the hollow sand specimens in comparison to the dense sand specimen over the hollow volume fraction.

		R_S_		
		No. 3	No. 4	No. 5
Compressive strength		1.64	2.73	1.74
Bending strength		1.12	1.96	1.21
High-temperature residual Tensile strength	298 K	1.75	1.52	1.40
	373 K	1.84	1.54	1.48
	473 K	1.78	1.42	1.42
	573 K	1.84	1.51	1.47
	673 K	2.15	1.67	1.80
	773 K	1.88	1.61	1.54

**Table 3:** The deformation and residual stress of the central thin rod in the three castings.

No.	Thin Rod Length (mm)	Linear Contraction (mm)	Residual Stress (MPa)
P1DN	148.50	−1.50	43.65
P2TN	148.20	−1.80	28.15
P3SN	148.25	−1.75	9.29

## Data Availability

Data are contained within the article.
